# YOLO-Act: Unified Spatiotemporal Detection of Human Actions Across Multi-Frame Sequences

**DOI:** 10.3390/s25103013

**Published:** 2025-05-10

**Authors:** Nada Alzahrani, Ouiem Bchir, Mohamed Maher Ben Ismail

**Affiliations:** 1 Computer Science Department, College of Computer and Information Sciences, King Saud University, Riyadh 11451, Saudi Arabia; obchir@ksu.edu.sa (O.B.); mbenismail@ksu.edu.sa (M.M.B.I.); 2 Computer Science Department, College of Computer Engineering and Science, Prince Sattam Bin Abdulaziz University, Al-Kharj 16278, Saudi Arabia

**Keywords:** action detection, keyframe extraction, fusion technique, spatiotemporal information, you only look once (YOLO)

## Abstract

Automated action recognition has become essential in the surveillance, healthcare, and multimedia retrieval industries owing to the rapid proliferation of video data. This paper introduces YOLO-Act, a novel spatiotemporal action detection model that enhances the object detection capabilities of YOLOv8 to efficiently manage complex action dynamics within video sequences. YOLO-Act achieves precise and efficient action recognition by integrating keyframe extraction, action tracking, and class fusion. The model depicts essential temporal dynamics without the computational overhead of continuous frame processing by leveraging the adaptive selection of three keyframes representing the beginning, middle, and end of the actions. Compared with state-of-the-art approaches such as the Lagrangian Action Recognition Transformer (LART), YOLO-Act exhibits superior performance with a mean average precision (mAP) of 73.28 in experiments conducted on the AVA dataset, resulting in a gain of +28.18 mAP. Furthermore, YOLO-Act achieves this higher accuracy with significantly lower FLOPs, demonstrating its efficiency in computational resource utilization. The results highlight the advantages of incorporating precise tracking, effective spatial detection, and temporal consistency to address the challenges associated with video-based action detection.

## 1. Introduction

Videos are a powerful medium for conveying emotions, actions, and physical characteristics owing to human sensitivity to visual information [1]. Their increasing popularity across domains, such as education [2], marketing [3], medical inspections [4], and video surveillance [5,6,7], is driven by advancements in intelligent devices, social networks, and communication technologies. However, the exponential growth of video data captured using personal cameras, CCTVs, and drones presents significant challenges. Processing this massive volume of data is computationally expensive, and manual analysis is impractical. For instance, an HD video recorded at 30 frames per second requires approximately 10 GB of memory per minute, emphasizing the need for automated solutions [8,9].

Unlike static images, videos consist of a sequence of frames over time (T × H × W), with temporal dynamics integral to understanding the actions and events. Deep learning approaches, particularly Convolutional Neural Networks (CNNs), have revolutionized visual data processing by automating feature extraction and achieving state-of-the-art performance in pattern-recognition tasks [10,11]. CNNs employ a hierarchical structure in which convolution and pooling layers extract low-to-high-level features, progressively capturing more complex patterns. For the video data, both 2D and 3D CNNs were employed. Whereas 2D CNNs operate independently on spatial dimensions, 3D CNNs extend operations to the temporal dimension, preserving motion information critical for action detection.

In video-based tasks, temporal relationships play a pivotal role in distinguishing similar actions, such as “sitting down” versus “standing up”. Single-frame CNN models classify each video frame independently but neglect the temporal connections between frames, leading to suboptimal performance [12]. To address this limitation, fusion techniques such as Early Fusion, Late Fusion, and Slow Fusion have been proposed [13,14,15,16]. These methods aggregate temporal information at different stages of the network, with Slow Fusion using 3D convolutions for progressive temporal feature integration. Although effective, these approaches can be computationally intensive.

Action detection, which is a core task in video analysis, involves identifying and localizing actions within video sequences. Unlike image-based recognition, video-based action detection must account for spatial and temporal dynamics and often requires advanced architecture. Traditional two-stage pipelines detect the actors and classify their actions in separate steps. Although straightforward, this approach introduces inefficiencies and suboptimal results owing to its sequential nature. End-to-end models address these issues by jointly optimizing actor detection and action classification but often demand significant computational resources [17].

Recent advancements in dependency modeling have further enhanced video understanding. Techniques such as non-local blocks [18], Long-term Feature Banks (LFBs) [19], and Squeeze-and-Excitation (SE) blocks [20] capture global relationships, long-term temporal dependencies, and channel-wise interdependencies. For example, non-local blocks leverage self-attention to model relationships across distant features, whereas LFBs provide memory mechanisms to relate present actions to past events. SE blocks dynamically prioritize relevant feature channels, enhancing the network’s ability to recognize complex patterns. These techniques collectively enable more robust video-based action-detection systems.

Building on recent advancements, this paper introduces a novel model for video-based action detection called the YOLO Act. It combines efficient feature extraction, robust dependency modeling, and end-to-end optimization. YOLO-Act is a new version of YOLOv8 that extends the capabilities of YOLOv8 to effectively recognize and classify movements. Specifically, this framework integrates keyframe extraction, action tracking, and class fusion to perform action detection tasks.

## 2. Related Work

Deep-learning models have recently advanced significantly in the domains of action classification and action detection, which, while closely related, address distinct challenges. Action classification focuses on recognizing and categorizing actions within a given video or sequence and treating the entire video as a single entity. In contrast, action detection involves recognizing actions and localizing them in both the spatial and temporal dimensions, making it a more complex task. Moreover, attention mechanisms have been integrated into deep-learning models, enabling a more focused representation of critical features by capturing intra-frame dependencies (relationships within a single frame) and inter-frame dependencies (relationships across sequential frames). In the following section, we delve into deep learning approaches for action classification and detection and highlight the evolution of attention-based models.

### 2.1. Action Classification

Previous methodologies for action recognition predominantly depended on manually designed features such as Cuboids [21], HOG3D [22], and Dense Trajectories [23,24]. These methods provide a foundation for understanding motion and appearance but are limited by their manual feature extraction processes. As deep learning advances, 3D convolutional networks have become the primary framework for action classification [7,8,9]. These models jointly process the spatial and temporal dimensions, which, while effective, introduce challenges in fully capturing the dynamic nature of motion.

To mitigate this constraint, two-stream designs [14] have been developed. Two neural networks were used to capture spatiotemporal information. The first network serves as the spatial path, focusing on capturing the environmental context of an action. The second network constitutes the temporal stream path, which captures pixel displacement across consecutive frames by processing the optical flow representation. However, the reliance on pre-computed optical flow complicates end-to-end learning and increases the computational overhead. Slow-fast networks [25] serve as an alternative by processing video streams at varying frame speeds, enabling a rapid pathway to acquire motion properties while assimilating spatial and temporal information via lateral connections.

While the aforementioned studies primarily focused on model architectures for action classification, another significant area of research investigated the detailed relationships between actors and objects [26,27,28,29]. For instance, ACAR [30] exploits explicit priors about actor relationships beyond their track identity to capture actor–context–actor relationships. This approach extracts actor–context features by pooling information within bounding box regions and learning higher-level interactions between actors.

The study in [12] adopted a 3D convolutional model to process spatial and temporal information. The model features a multiresolution architecture designed to handle local motion information in videos. Specifically, it incorporates two streams: a context stream for modeling low-resolution image features and a fovea stream for processing high-resolution image features. Additionally, Early, Late, and Slow fusion techniques have been explored to combine temporal information. The experimental results indicate that the proposed model outperforms the single-frame baseline approach, albeit only slightly. However, this method has notable limitations: it is computationally expensive because of the use of 3D convolution filters and fails to account for the sequential order of frames.

To address these challenges, recent developments have introduced lightweight action recognition networks such as MobileNet-Action [31] and TinyVideoNet [32], which are designed for resource-constrained environments. These models prioritize computational efficiency while maintaining reasonable accuracy. Integrating lightweight backbones with object detectors like YOLO has become an attractive approach for real-time action recognition applications. Such integration typically involves feature sharing or sequential pipeline designs where object localization (YOLO) informs the action classification stage, thus balancing speed and performance.

In addition, efficient keyframe selection strategies play a critical role in video-based action recognition. Techniques range from heuristic approaches (e.g., selecting frames based on motion energy) to deep learning methods that predict informative frames [33,34]. Additionally, the use of tracking mechanisms improves temporal modeling by maintaining consistent object identities across frames, enabling richer temporal feature aggregation [35].

Alternatively, frame-level models with late fusion have been employed [36]. In this approach, a single frame is input into the model at a time, and the resulting frame-wise spatial representations are subsequently aggregated. This aggregation is achieved using CNNs combined with either temporal feature pooling or Recurrent Neural Networks (RNNs) [37], such as Long Short-Term Memory Networks (LSTMs) [38]. Although RNNs offer the advantage of learning spatiotemporal properties, the primary drawback of this approach lies in its computational complexity.

Furthermore, class imbalance, common in action recognition datasets, adversely affects model performance. Solutions include re-weighting loss functions [39], over-sampling minority classes, under-sampling majority classes, and generating synthetic data through adversarial techniques [40].

### 2.2. Action Detection Without Attention Mechanism

Several approaches have been proposed to integrate spatial and temporal information for action detection, each of which addresses different challenges in extracting meaningful features from video data. Faster R-CNN-based methods, such as those presented in works [6,41], process frames individually by extracting feature maps using ResNet [42] and generating region proposals to predict the object classes and bounding boxes. These methods effectively handle spatial information but process each frame independently, limiting their ability to capture temporal dynamics.

To address the integration of spatial and temporal features, the Granulated R-CNN (G-RCNN) [13] enhances real-time detection by combining temporal granules derived from frame differences to capture motion information with spatial granules segmented based on pixel similarity to capture spatial context. These granules are processed using AlexNet [43], enabling a more granular and efficient approach for action recognition and object detection.

The Watch Only Once (WOO) approach [44] adopts a different strategy by utilizing a Feature Pyramid Network (FPN) with ResNet to extract keyframes and employs an Actor Localization Head to predict bounding boxes. This method aggregates spatial and temporal embeddings to classify actions and ensure efficiency and accuracy.

Moreover, emerging research on detection in complex environments has yielded valuable insights applicable to action recognition. For instance, hybrid learning architecture proposed for high-speed railroad scene parsing [45] demonstrates efficient fusion of spatial and semantic information. Similarly, the RailFOD23 dataset [46] introduces challenges in foreign object detection under adverse conditions, emphasizing robustness. Additionally, Radiance Field Learners [47] explore 3D spatial awareness in UAV first-person view contexts, offering novel perspectives for dynamic scene understanding.

Alternatively, X3D [48] introduces a scalable spatiotemporal neural network designed for video action recognition that encompasses both classification and detection tasks. This approach uses a progressive network expansion strategy, incrementally transforming a compact 2D architecture into a 3D architecture. Key dimensions, such as temporal duration, spatial resolution, and network depth, were optimized to balance the performance and computational cost.

### 2.3. Attention-Based Approaches

Recent advancements in transformer architecture have significantly catalyzed progress in action recognition, with extensive research exploiting transformer-based backbones and self-attention mechanisms to push field boundaries [9,49,50,51,52]. In this context, Multiscale Vision Transformers (MViTs) [49] have established a hierarchical architecture to enhance dense prediction tasks such as action detection and segmentation. Building on this foundation, MViTv2 [50] introduces further feature representation and scaling improvements. Additionally, Video Masked Autoencoders (VideoMAE) [51] introduced a dual-masking strategy in both the encoder and decoder self-supervised video, drawing inspiration from ImageMAE [53].

To address the challenges of long-form video understanding, an Object Transformer [54] was introduced to model long-term temporal dependencies, thereby facilitating progress in comprehending complex video content. This study underscores the importance of capturing intricate temporal relationships to advance action recognition in extended video sequences.

Recent advancements in action recognition have led to a shift toward models that efficiently integrate spatial and temporal data to improve performance across various benchmarks. Among these, the Agent–Environment Network (AEN) [55] introduces a dual-pathway design: one pathway focuses on active agents using a transformer encoder, while the other models agent–environment interactions. These pathways are contextually fused using attention mechanisms, allowing the AEN to effectively capture agent-specific and contextual dynamics.

Similarly, the You Only Watch Once (YOWO) model [56] combines spatial and spatiotemporal features for action recognition. It extracts spatial features using Darknet-19 [57] and spatiotemporal features using 3D-ResNext-101 [58]. These features are aggregated via a Channel Fusion and Attention Mechanism (CFAM), whereas the YOLO-based regression module predicts bounding boxes. Together, these methods underscore the importance of integrating the spatial and temporal data.

In addition to these innovations, recent efforts have incorporated 3D pose estimation and tracking to further improve spatiotemporal modeling. For example, the Lagrangian Action Recognition Transformer (LART) [59] leverages human motion trajectories derived from 3D pose data along with contextual appearance features. To model relational dynamics, LART introduces multiperson interaction modeling using identity-preserved tracklets. While effective, LART’s reliance on robust tracking algorithms, such as PHALP [60], poses challenges in occlusion or rapid-motion scenarios.

In parallel, Hiera [61], a hierarchical vision transformer, adopts a different approach by streamlining the architecture of modern transformers. Unlike traditional methods that incorporate complex, vision-specific enhancements (often called “bells and whistles”) to boost supervised classification performance, Hiera demonstrated that such complexity is unnecessary. Instead, it utilizes masked autoencoding (MAE) as a pretraining visual pretext task, showing that the spatial biases required for vision tasks can be learned through effective pretraining. Table 1 provides an overview of reported action classification and action detection approaches.

### 2.4. Discussion

To incorporate temporal information, most related works employ a 3D CNN [16]. These models consider spatial-temporal connections between consecutive frames through 3D spatial-temporal processing over a set of frames. However, they exhibit high time complexity, and capturing long contexts and time dependencies is challenging. Moreover, Faster R-CNN [62], initially designed for object detection within images, is generally integrated into the contextual path to pool the region of interest, including the actor. Nevertheless, expanding the actor bounding boxes to accommodate more surrounding context information improves the spatiotemporal action detection [63]. In fact, the features extracted from the entire frame preserve more spatial details than the pooled ROI.

Moreover, traditional methods like Cuboids, HOG3D, and Dense Trajectories rely heavily on hand-crafted features and predefined motion patterns, limiting their adaptability to complex real-world scenarios. In contrast, YOLO-Act leverages deep learning to automatically learn discriminative spatial features (e.g., pose, interaction) and temporal dynamics (e.g., motion trajectories) from data. This results in superior generalization across diverse action categories, complex interactions, and varied motion speeds. Table 2 summarizes the advantages of deep learning approaches compared with manual methods.

**Table 1:** Overview of deep learning algorithms for action detection.

Task	Reference	Input	Model	Dataset	Performance
Action Classification	[12]	Raw frames	3D CNN	Sports-1M [12]	Hit@1 = 60.9%
	[36]	Raw Frames Optical Flow	CNN LSTM	Sports-1M [12] UCF-101 [64]	Hit@1 = 73.1% 3-fold Acc = 88.6%
	[14]	Single Frame Optical Flow	Two-stream CNN	UCF-101 [64]	Mean Acc = 88.0–59.4%
	[25]	Raw frames (low rate and fast rate)	SlowFast	AVA [63]	mAP = 23.8
	[30]	Keyframe	ACAR	AVA [63]	mAP = 33.3
Action Detection without attention mechanism	[6]	Single frame	Faster R-CNN Binary classifier	Prepared their own DS	Precision = 93.87%
	[44]	Keyframe	WOO, SlowFast	AVA [63] JHMDB [65]	mAP = 28.3 mAP = 80.5
	[41]	Single frame	CNN	Prepared their own DS	AP50 = 95.20
	[13]	Keyframes	Faster R-CNN AlexNet deep SORT	Prepared their own DS	mAP = 80.6
	[48]	Raw Frames	X3D	AVA [63]	mAP = 27.4
Attention-Based Approaches	[55]	Keyframes Raw frames	SlowFast Faster R-CNN	ActivityNet [66] THUMOS [67]	AR@AN = 68.99–64.03
	[56]	Keyframes Raw frames	2D CNN 3D CNN	JHMDB [65] UCF101 [64]	mAP = 64.9–79.2
	[54]	Key frames	Object transformer	AVA [63]	mAP = 31.0
	[49]	Raw frame	MViT v1	AVA [63]	mAP = 28.7
	[50]	Raw frame	MViT v2	AVA [63]	mAP = 31.6
	[59]	3D human pose data and tracking information	LART	AVA [63]	mAP = 45.1
	[61]	Raw frames	Hiera	AVA [63]	mAP = 43.3

Late fusion is generally adopted to aggregate the results of single frames or to combine the results of contextual and temporal features. Specifically, the final video representations learned by CNN deep learning models are commonly generated via global average pooling. Essentially, it computes the first-order statistics of convolution features over the temporal dimension, ignoring the richer statistical information included in spatiotemporal features and having a limited ability to capture the complicated dynamics of video frames.

Attention methods are alternative solutions that focus on essential frames and regions by capturing the spatial and temporal frame relationships. Specifically, attention modules are based on temporal covariance pooling to characterize intra-frame correlations and inter-frame cross-correlations. These are commonly integrated into transformers. In addition, attention mechanisms allow models to dynamically focus on salient actors or action-relevant frames, leading to improved detection performance, especially in crowded or cluttered scenes. However, training a transformer model from scratch requires a large amount of data [10,54]. Attention-based and attention-less techniques for action recognition are compared in Table 3.

## 3. The Proposed YOLO-Act Model

Existing deep learning models have proven to be effective in addressing the challenging problem of spatiotemporal action detection. However, several barriers limit the capabilities of these models. Namely, (i) the extraction and integration of spatiotemporal information and (ii) the localization of the actor without omitting the environmental context remain open challenges that constrain the deep learning models used for video-based action detection.

**Figure 1:**
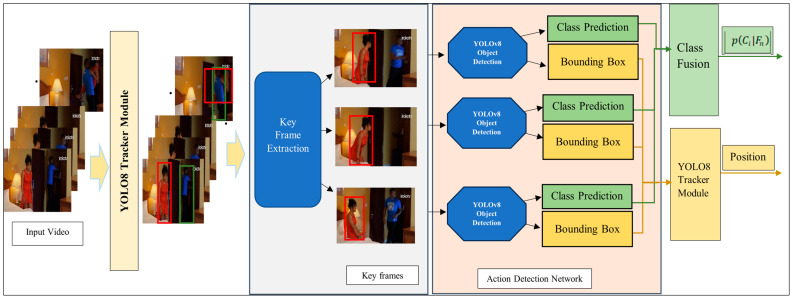
Block diagram of the proposed approach.

Accordingly, we propose a novel approach to alleviate spatiotemporal action-detection challenges. The proposed human action detection approach utilizes an integrated detection and tracking network along with a keyframe extraction module, as shown in Figure 1.

At the core of this framework is the YOLOv8 [68]. YOLOv8, one of the latest versions of YOLO (You Only Look Once), is renowned for its improved detection accuracy, faster inference speeds, and optimized architecture that balances precision and computational efficiency. These improvements are driven by innovations, such as adaptive anchor-free detection, dynamic label assignment, and an upgraded backbone network. Additionally, its flexibility to handle varying input resolutions, capacity to process frames with high precision, and streamlined architecture make it an ideal baseline candidate for extension to action recognition challenges, including spatiotemporal complexities.

As such, YOLOv8, initially designed for single-frame object detection, was employed as the baseline for developing the new action recognition model, YOLO-Act. Specifically, the proposed approach extends YOLOv8 capabilities to handle spatiotemporal dynamics, enabling it to perform action recognition. By utilizing YOLOv8’s strong foundation and introducing enhancements incorporating temporal analysis and motion modeling, YOLO-Act bridges the gap between static object detection and dynamic activity recognition. Figure 1 illustrates the architecture of the proposed approach.

Furthermore, YOLO-Act extends YOLOv8 by embedding temporal awareness, enabling it to model motion cues over time. This design is critical for recognizing dynamic human actions, where context evolves between frames, which static object detectors cannot capture effectively. Table 4 presents a comparison between YOLOv8 and the proposed YOLO-Act.

### 3.1. Keyframe Extraction

The number of frames processed affects the scalability and usability of the action detection approach. Moreover, the number of frames is a dilemma. Although one frame benefits the actor’s localization, it is insufficient for action classification. The latter requires several frames to extract temporal information. However, too many keyframes can be counterproductive. Furthermore, the choice of frames affects the performance of the detection model. The frames should not be redundant; rather, keyframes that exhibit the main changes in the video should be employed.

We adopt a first–middle–last frame selection strategy for keyframe extraction. Specifically, the first frame corresponds to the initial frame of the detected action window, while the last frame represents the final frame of the action window. The middle frame is defined as the frame located at the temporal center, i.e., halfway between the first and last frames. For actions of varying durations, the middle frame is dynamically computed based on the midpoint between the starting and ending frame indices, ensuring that the temporal structure of the action is consistently represented regardless of its length.

As mentioned above, in YOLO-Act, only three frames (the first, middle, and last frames) per action are deliberately selected to represent the action’s temporal evolution. This strategy captures the critical phases of an action: the initiation, the peak (or steady state), and the conclusion. Despite accessing a limited number of frames, this approach preserves sufficient temporal dynamics to enable effective action recognition. The choice of using only three frames was guided by a trade-off between computational efficiency and predictive performance. Processing fewer frames significantly reduces inference time, memory usage, and computational complexity, making YOLO-Act highly suitable for real-time applications. In fact, by focusing on the most informative moments of an action, YOLO-Act avoids the redundancy often present in video frames, thus enabling the model to learn more discriminative temporal features without being overwhelmed by irrelevant or repetitive information. Furthermore, key actions in many practical scenarios can be sufficiently characterized by their start, mid-point, and end dynamics, making the three-frame strategy particularly effective for most action types.

In summary, although various techniques aim to distill a video into a representative set of frames, the first–middle–last strategy provides distinct advantages. First, it is simple and efficient. By selecting keyframes at the beginning, middle, and end of an action, essential scenes that encapsulate the main content and transitions within the action are captured. Second, the first–middle–last keyframe extraction method maintains temporal continuity, ensuring that the selected frames consistently represent the action. Third, the first–middle–last approach is more resistant to outliers and noisy data, as it explicitly targets frames at the beginning, middle, and end of the video, which are likely to be the most information-rich.

### 3.2. Action Detection

Human action detection involves classifying an action and localizing the actor in each frame. Nevertheless, localizing the actor should not neglect the environmental context, which is useful for classification. Similarly, the environmental context should not obstruct action detection by overfitting the pattern to the context considered. In this regard, we propose to employ the You Look Only Once YOLOv8 [68] object detection model for object detection in each frame. It considers the environmental context while recognizing an object of interest. Contrary to region-based approaches, such as Faster R-CNN [62], it does not extract the region of interest and pool it from the environmental context before conveying it to the classification module.

**Figure 2:**
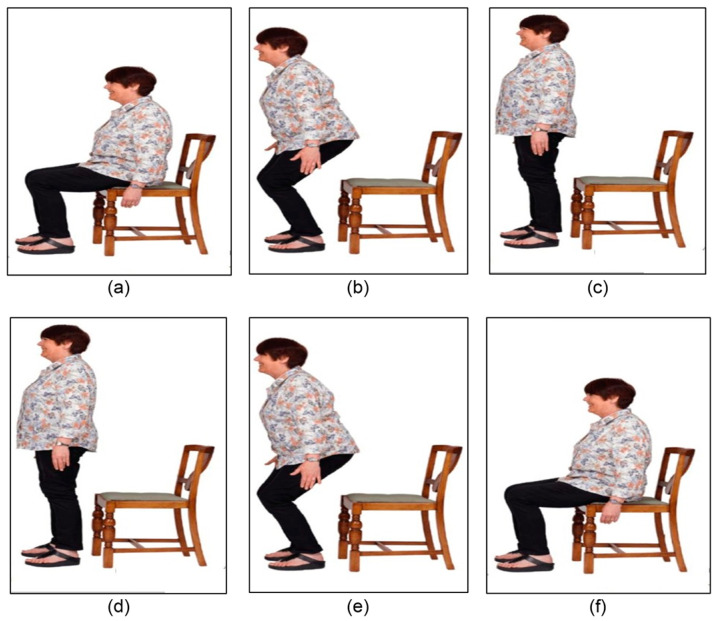
Two illustrative examples of action sequences. (**a**) starting of the action stand up, (**b**) middle of the action stand up, (**c**) end of the action stand up, (**d**) starting of the action sit down, (**e**) middle of the action sit down, and (**f**) end of the action sit down.

The frame order should be incorporated to exploit temporal information. For this purpose, each frame was labeled using an action label and the order of occurrence in the video. For example, if three keyframes are extracted from a video representing the action “Standing up”, the first frame will be labeled “Standing up 1”, the second frame “Standing up 2”, and the third one “Standing up 3”. Figure 2 displays two illustrative examples where three frames are extracted from the action “Standing Up” and the action “Sitting down”. As can be seen, the first frame of the action “Standing up” (Figure 2a) is similar to the third frame of the action “Sitting down” (Figure 2f); they are labeled differently as “Standing up 1”, and “Sitting down 3”. Accordingly, the YOLOv8 model was trained for each order. Specifically, the first YOLOv8 model used the first shot of each action and the bounding box surrounding the actor to predict the start of each action. Similarly, for the subsequent action shots, the YOLOv8 s and third models were trained to predict the middle and end action shots, respectively.

### 3.3. Class Fusion

Let $p\left(C_{in}|F_{n}\right)$ denote the probability that the action keyframe $F_{n}$ is the $n^{th}$ action keyframe of action i. It is predicted by the $n^{th}$ YOLOv8 model as the confidence score of $F_{n}$ with respect to $C_{in}$. Similarly, let $p\left(C_{i}|\left(F_{1},F_{2},\ldots ,F_{N}\right)\right)$ be the probability that the action represented by the N consecutive frames $\left(F_{1},F_{2},\ldots ,F_{N}\right),$ belongs to class $C_{i}$.

To classify the actions, we intend to employ a Late Fusion technique [12]. Subsequently, the confidence scores from individual keyframes are combined using a multiplicative rule. Specifically, the final probability $p\left(C_{i}|\left(F_{1},F_{2},\ldots ,F_{N}\right)\right)$ that an action sequence belongs to class $C_{i}$ is computed as the product of the individual keyframe probabilities $p\left(C_{in}|F_{n}\right)$, as shown in Equation (1). This approach assumes that the predictions for each keyframe are conditionally independent, and it emphasizes classes that are consistently supported across all keyframes. Formally,

(1) $$p\left(C_{i}|\left(F_{1},F_{2},\ldots ,F_{N}\right)\right)=\prod_{n=1}^{N}p\left(C_{in}|F_{n}\right)$$

This multiplicative fusion strategy enhances robustness by reducing the influence of isolated misclassifications: if a keyframe incorrectly predicts an action class with low confidence, its effect on the overall prediction becomes minimal. Consequently, only action classes consistently supported across multiple keyframes achieve a high final confidence score.

### 3.4. Actor Tracking

The YOLOv8 tracker was employed to track the action from beginning to end. More specifically, it is used to associate detected actors across frames using a combination of appearance embeddings and motion prediction via a Kalman filter. In cases of multiple actors moving closely or overlapping, the tracker resolves ambiguities through intersection-over-union (IoU) matching with a threshold of 0.3 and appearance similarity scoring. To minimize identity switches, a re-identification mechanism is applied using actor-specific visual features extracted from bounding boxes. Subsequently, the first, middle, and last frames were extracted. Moreover, to ensure that the actor is accurately localized across all frames, the tracker component of the model is employed to propagate detections from the keyframe to subsequent frames. Specifically, it tracks the bounding box of a detected actor as it moves between consecutive frames.

## 4. Experiments

Experiments were conducted to evaluate the effectiveness of the proposed YOLO-Act model as a unified CNN architecture for spatiotemporal human–action detection.

### 4.1. Dataset Description

YOLO-Act was evaluated using the AVA dataset [63], which is a benchmark dataset designed for action detection. This dataset focuses on localizing individuals within spatiotemporal volumes and assigning action labels to them. It is important to note that this action detection benchmark dataset is pre-structured into three major sub-datasets: person movement, person–object manipulation, and person-person interaction. This standard division is widely adopted by state-of-the-art action detection methods [59,63] to ensure consistent and comparable evaluations across different models. Following this established protocol allows us to benchmark YOLO-Act fairly against existing approaches and ensures that our evaluation remains aligned with recognized practices in the action detection community.

The AVA dataset contains approximately 430 video clips, each 15 min long, annotated at 1Hz. It covers 80 atomic action classes with an average of 2.5 labels per person per frame. Each actor is labeled with one primary action and optional annotations for up to three person–object interactions and three person–person interactions. The data distribution is highly imbalanced; for instance, common actions like “standing” and “talking” dominate the dataset, while rare actions such as “playing musical instruments” are under-represented. To address this, we employed a weighted cross-entropy loss to give higher importance to minority classes during training.

**Figure 3:**
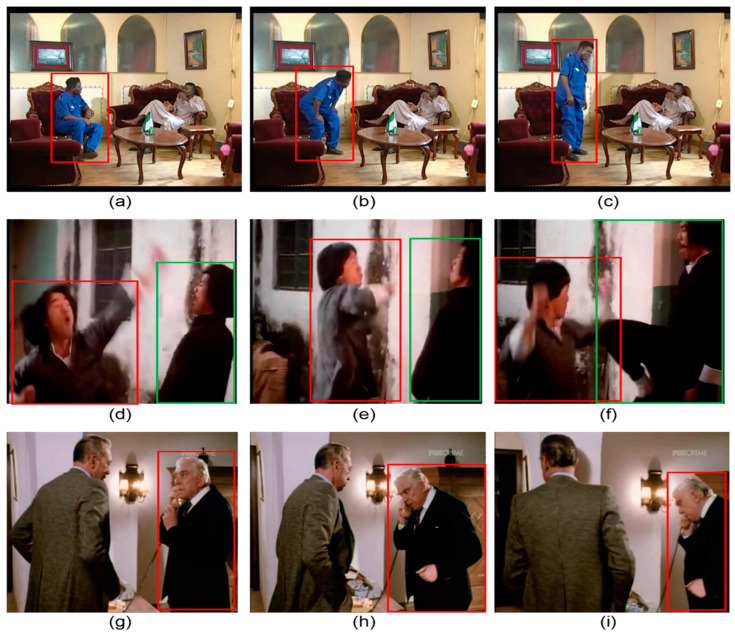
Sample action keyframes and corresponding labeling. (**a**) Beginning of action stand, (**b**) middle of action stand, (**c**) end of action stand, (**d**) beginning of person-to-person action fight, (**e**) middle of person-to-person action fight, (**f**) end of person-to-person action fight, (**g**) beginning of person-to-object action answer phone, (**h**) middle of person-to-object action answer phone, (**i**) end of person-to-object action answer phone.

For each action, the first, middle, and last frames were extracted and labeled as the beginning, middle, and end of the action, respectively. Ground-truth bounding boxes are provided to localize the actor and/or action. Thus, for each action, three keyframes and the actor’s coordinates (along with the coordinates of the object, if applicable) were considered. Figure 3 illustrates the three sample actions and their corresponding labeling approaches. Figure 3a–c depict the person’s action “Stand”, with three action labels: stand_1 (beginning of the action), stand_2 (middle of the action), and stand_3 (end of the action). Each frame includes a bounding box surrounding the person performing the action. Similarly, Figure 3d–f present a sample of a person-to-person interaction labeled as “fight/hit (a person)”. Finally, Figure 3g–i represent a person-to-object action. In this case, the bounding boxes include both the person and the object involved in the action.

Each sub-dataset is further divided into three smaller subsets: one representing the beginning of the movement called “Starting frames”, another capturing the middle of the movement called “middle frames”, and the last focusing on the end of the movement called “ending frames”.

### 4.2. Experiment Setting and Training

The experiments were conducted on a system running Windows 11 equipped with 64GB RAM, an NVIDIA GeForce RTX 4080 SUPER GPU, and an Intel i9-14900F CPU, sourced from Newegg (Newegg Inc., City of Industry, CA, USA). The software platform consisted of Torch 2.4.0 with CUDA 12.6, managed by Anaconda3. The dataset was divided into 60% for training, 20% for validation, and 20% for testing.

As outlined in Section 3, the proposed YOLO-Act framework integrates three deep-learning models based on the YOLOv8 architecture. These models were pretrained on the COCO dataset [69] before being fine-tuned on specific subsets of the dataset. The first model is trained using the training set of the “starting frames” sub-dataset, the second on the “middle frames” sub-dataset, and the third on the “ending frames” sub-dataset. All action categories, including person movement, person–object manipulation, and person-person interaction, were utilized to train each sub-model.

Table 5 summarizes the training configurations used in this study. Several regularization techniques have been employed to enhance performance and generalization. These included a dropout rate of 0.1 and early stopping with a patience of 10 epochs. Furthermore, a warm-up phase spanning three epochs was introduced to stabilize the initial stages of training. A cosine decay learning rate schedule was applied to gradually reduce the learning rate, enabling a more effective fine-tuning of the models.

**Figure 4:**
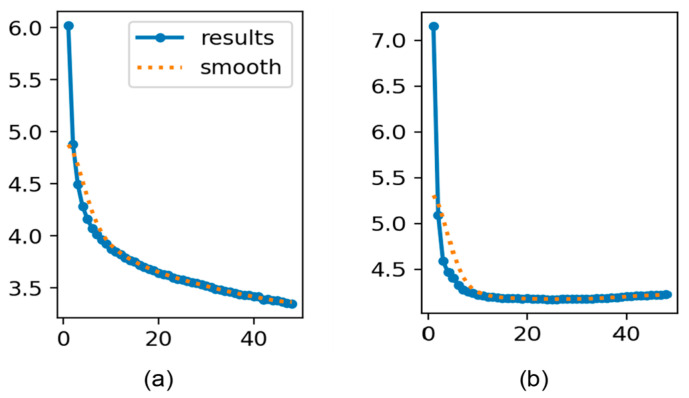
Training and validation classification loss trends across epochs. (**a**) Classification loss during training and (**b**) validation classification loss.

These three models converged after approximately 40 epochs. Figure 4 depicts the classification loss trends during training, highlighting a steady decline followed by stabilization in both the training and validation sets. This pattern indicates effective learning with a minimal risk of overfitting. These measures result in a lightweight model that balances complexity and performance and enhances its ability to generalize effectively. Consequently, the model demonstrated robust performance on unseen data, further corroborated by the achieved performance metrics.

### 4.3. Testing YOLO-Act

A testing dataset was used to test the model. In this phase, the ground truth labels for the three keyframes (start, middle, and end) are not used, nor are the bounding boxes provided to the three sub-models. Instead, the models predict both the action category and the bounding boxes. Specifically, a video segment is first processed using a pre-trained YOLOv8 model that incorporates a tracking component to identify and track individuals. The start, middle, and end frames are extracted from the tracked segments. Each extracted frame is then passed to its respective trained sub-model for prediction. Equation (1) in Section 3 detailed the combined confidence scores obtained from the three sub-models to determine the final action category. The bounding boxes predicted by each sub-model are used to localize the action and are further evaluated by computing the mean average precision (mAP) and Floating-Point Operations per Second (FLOPs). The results obtained were compared with those of state-of-the-art approaches to assess the effectiveness of the proposed method.

### 4.4. Experimental Results Analysis

The proposed YOLO-Act model achieves 73.28 mAP while significantly reducing computational complexity, cutting up to 90% of GFLOPs compared with existing methods. Table 6 compares YOLO-Act with other state-of-the-art methods.

Overall, YOLO-Act has a gain of +28.18 mAP compared with the second-best-performing model, LART [49]. These results demonstrate that the proposed YOLO-Act model outperforms the LART model based on 3D pose estimation for action recognition on the AVA dataset.

This improvement could be attributed to several factors. First, YOLOv8 provides precise spatial localization using highly accurate bounding boxes and confidence scores, which are crucial for downstream action recognition tasks. The inclusion of a tracking component ensures temporal consistency by linking bounding boxes across frames, reducing noise, and enhancing contextual understanding of ongoing actions.

Furthermore, the class fusion strategy leverages multiple predictions to refine the classification results and effectively integrates spatial and temporal features. In contrast, the LART model relies heavily on 3D pose information, which can be noisy or incomplete in complex scenes with occlusions or crowded environments such as those in the AVA dataset. Additionally, YOLO-Act balances spatial and temporal learning, making it better suited to AVA’s annotation structure, where sparse temporal labeling may limit the efficacy of pose-based models. These findings highlight the effectiveness of combining object detection, tracking, and class fusion for action recognition, particularly in challenging pose-estimation scenarios.

Another key distinction of the YOLO-Act is its explicit association with individuals over time through tracking, which most current action recognition methods do not incorporate. Existing approaches typically rely on mid-frame bounding boxes to predict an action, which may fail to capture the full temporal context of an individual’s movement. For instance, when a person enters a scene, a single cropped frame often lacks sufficient information to represent an entire action. Tracking addresses this limitation by maintaining the position of an individual across multiple frames, thereby providing more comprehensive and localized temporal information. This enhanced temporal understanding allows the model to make more accurate predictions and highlights the critical role of tracking in action-recognition tasks.

To evaluate YOLO-Act’s performance against leading action detection frameworks, we conducted a comparative analysis with SlowFast Networks R101 [25] and I3D [70], two widely recognized baselines. As shown in Table 6, YOLO-Act achieves a substantial improvement in detection accuracy, with a relative mAP increase of 51.38% over I3D and 49.48% over SlowFast. Additionally, YOLO-Act maintains lower computational complexity (114.41 GFLOPs versus 138 GFLOPs for SlowFast), reinforcing its suitability for real-time applications. These gains stem from YOLO-Act’s unified and streamlined architecture, which integrates action localization and classification within a single forward pass, unlike multi-stage models that require separate processing for spatial and temporal cues. Central to this efficiency is YOLO’s dense grid-based prediction mechanism, which divides the image into a grid where each cell independently predicts bounding boxes and class probabilities. This design enables simultaneous, parallel predictions across the entire frame, ensuring both high speed and full spatial coverage. Built on this foundation, YOLO-Act extends the grid-based approach to action detection by directly predicting actor locations and action classes per cell, without relying on region proposals or optical flow. As a result, it (i) detects multiple actions in real-time, (ii) minimizes redundant computations, (iii) maintains spatial awareness, and (iv) remains computationally lightweight compared with 3D CNNs and two-stream models. Furthermore, by incorporating temporal aggregation into this efficient framework, YOLO-Act captures fine-grained motion patterns without sacrificing inference speed. Its compact design enhances robustness across diverse video scenarios and reduces dependency on external modules. In other words, these advantages underscore YOLO-Act’s strong balance between accuracy, speed, and efficiency, making it well suited for practical, real-world action detection deployments.

Moreover, we assessed the real-time performance of YOLO-Act by measuring its inference speed in frames per second (FPS) on an NVIDIA GeForce RTX 4080 SUPER GPU. YOLO-Act achieved an average processing speed of 64.79 FPS, significantly outperforming TubeR [71] at 40 FPS and ACAR-Net [30] at 48.7 FPS. This high inference speed highlights YOLO-Act’s efficiency and confirms its suitability for real-time, low-latency action detection in practical applications.

To better understand the performance of YOLO-Act, Table 7 compares it with the second-best-performing approach, LART [59], with respect to the three AVA subcategories: Object Manipulation (OM), Person Interactions (PI), and Person Movement (PM).

For the person-movement task, which includes actions such as running, standing, and sitting, the YOLO-Act model achieves 67.1 mAP, outperforming LART, which achieves 63.8 mAP. The YOLO-Act model demonstrated significant advantages over LART [59], particularly in the PM subcategory of the AVA dataset. Unlike LART, which relies heavily on static 3D pose estimation of the middle frame, YOLO-Act performs very well in both spatial and temporal modeling. Specifically, using YOLOv8’s highly accurate bounding box prediction, the model ensures the precise localization of individuals performing actions. Moreover, tracking enhances temporal consistency by associating people across frames, capturing the progression of movements more effectively, and minimizing the errors caused by frame-to-frame variations. Furthermore, the proposed fusion approach efficiently aggregates this information to predict the movement of a person accurately.

**Figure 5:**
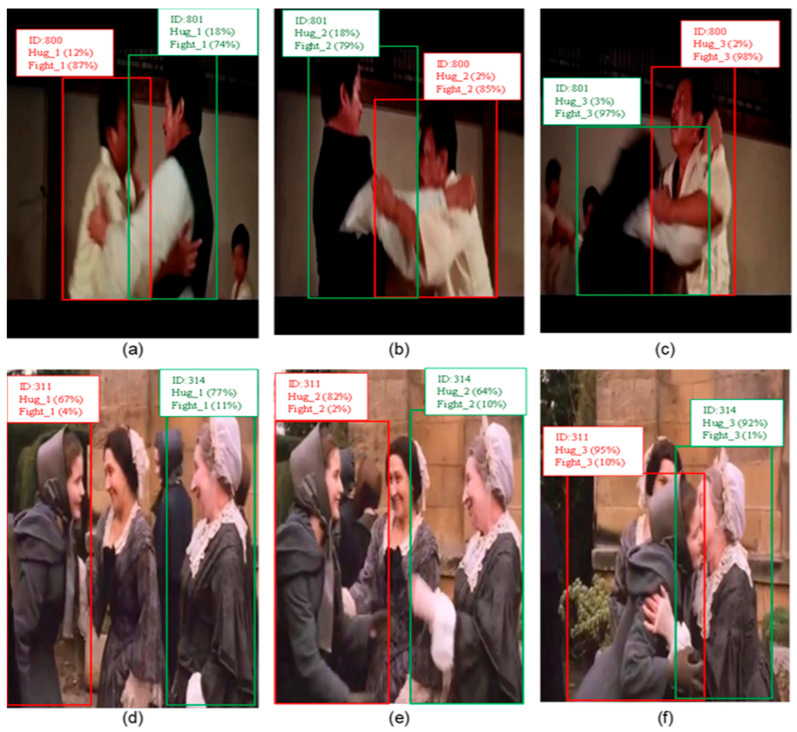
Predictions generated by YOLO-Act for the actions fight and hug, each involving two interacting individuals. Bounding boxes are shown around both persons, labeled with person ID, predicted action, temporal stage, and confidence score. Red and green boxes distinguish the participants. (**a**) Start of the action fight, (**b**) middle of the action fight, (**c**) end of the action fight, (**d**) start of the action hug, (**e**) middle of the action hug, (**f**) end of the action hug.

Similarly, YOLO-Act excels in the person–person interaction category, achieving 74.9 mAP, significantly surpassing LART’s 45.1 mAP. This can be explained by the YOLO-Act employing a track-then-detect approach, where the person is first tracked, and then bounding boxes are predicted with high precision and fused across frames. This ensures accurate localization and provides a solid foundation for tracking individuals in interactions involving multiple people. Figure 5 highlights the capability of YOLO-Act to effectively capture complex interactions and distinguish between visually similar actions, such as “fight a person” and “hug a person”. Figure 5a–c show three consecutive frames related to the “fight” action, with confidence scores reported for both “hug” and “fight” actions.

In addition, the tracking component assigns consistent IDs to individuals, thereby ensuring accurate tracking across frames. The same ID was correctly assigned to each person in all frames. Notably, throughout the sequence, the confidence score for the “ fight “ action remains higher than that for the “hug”, even in potentially ambiguous scenes, such as in Figure 5a. This accuracy was achieved by employing contextual information for the action prediction. A similar analysis applies to the “hug a person” action depicted in Figure 5d–f. The individuals were effectively tracked across frames with consistent IDs. Moreover, the confidence score for “hug” consistently surpasses “ fight “ across all three frames, reflecting the ability of the YOLO-Act to accurately identify the action based on contextual and temporal information.

Another critical factor is the YOLO-Act’s three connected frames to represent each action, enabling the model to incorporate the temporal context and capture the dynamic progression of interactions. This temporal granularity is significant for person–person interactions, where actions unfold over time. By employing this design, the model can better differentiate subtle or overlapping interactions, such as distinguishing between hugging and fighting. However, LART’s reliance on single-frame pose-based detection might lack the temporal resolution necessary to fully capture these nuances. These methodological differences allow YOLO-Act to significantly outperform LART, with notable gains such as +15.4 mAP in fighting and a relative improvement of over +217% in hugging.

However, applying YOLO detection without any temporal aggregation led to a 64.88% performance drop, highlighting the importance of modeling temporal context. These results, summarized in Table 8, demonstrate that temporal aggregation modules significantly enhance overall system performance.

The substantial improvement of the YOLO-Act in the Object Manipulation (OM) category, achieving 76 mAP compared with LART’s 37.3 mAP, underscores the importance of explicit object modeling. Unlike LART, which does not explicitly model objects and relies primarily on pose information, YOLO-Act adopts YOLOv8’s high-precision object detection capabilities to localize not only the person but also the objects involved in the interactions. This explicit object detection allows the model to associate the person with the manipulated object, providing a crucial context for recognizing actions, such as answering a phone, carrying an object, or eating. Moreover, the track-then-detect approach ensures consistent and accurate associations between a person and an object across multiple frames. By tracking the individual and the object over time, YOLO-Act captures their dynamic relationship, which is essential for understanding object manipulation actions. By contrast, LART’s 3D object modeling may lead to incomplete contextualization of such interactions, resulting in significantly lower performance in this category.

Additionally, YOLO-Act’s use of three connected frames provides a richer temporal context, allowing it to capture the progression of object manipulation actions more effectively. This design enables the model to distinguish between similar poses, such as reaching for an object versus holding it, based on the temporal evolution. These methodological strengths collectively highlight the robustness of YOLO-Act in capturing and interpreting object manipulation actions, setting it apart from LART and resulting in a significant improvement in mAP.

**Figure 6:**
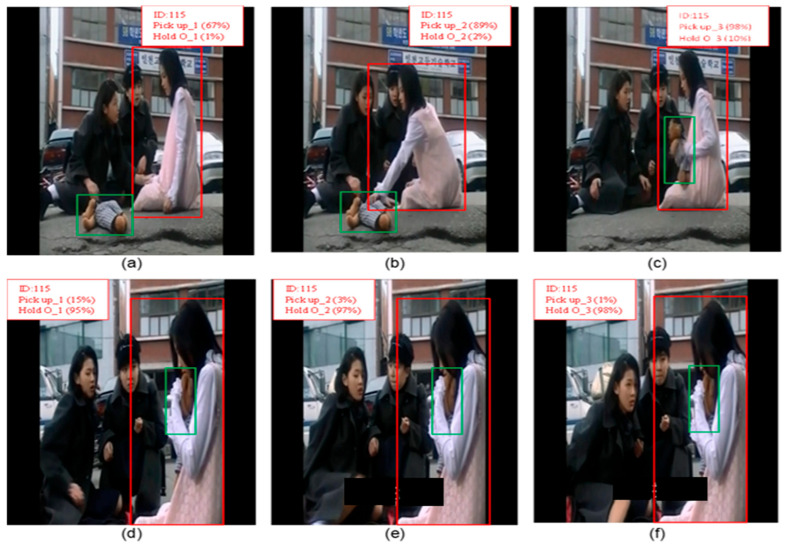
Illustration of YOLO-Act’s predictions of Object Manipulation (OM) category for the actions pick up and hold (O), each involving an interacting individual and object. Bounding boxes are shown around the person, labeled with person ID, predicted action, temporal stage, and confidence score. Red and green boxes distinguish the participants. (**a**) start of the action pickup, (**b**) middle of the action pickup, (**c**) end of the action pickup, (**d**) start of the action hold (O), (**e**) middle of the action hold (O), (**f**) end of the action hold (O).

Figure 6a,c illustrates three consecutive frames showing the “lift/pick up” action, with confidence scores presented for both the “lift/pick up” and “carry/hold (an object)” actions. By analyzing three connected frames, YOLO-Act effectively utilizes an enriched temporal context to capture the sequential progression of object manipulation. Throughout the sequence, the confidence score for the “lift/pick up” action consistently remains higher than that of “carry/hold”, even when the poses appear similar, as shown in Figure 6d. This level of precision is achieved through the model’s ability to interpret action dynamics over time. A comparable observation is made for the “carry/hold (an object)” action depicted in Figure 6d–f. By detecting objects within the context of three connected frames, the model can link the person with the object being manipulated, offering an essential context for differentiating between closely related actions. As demonstrated, the confidence score for “carry/hold” consistently surpasses “lift/pick up” across all three frames.

**Figure 7:**
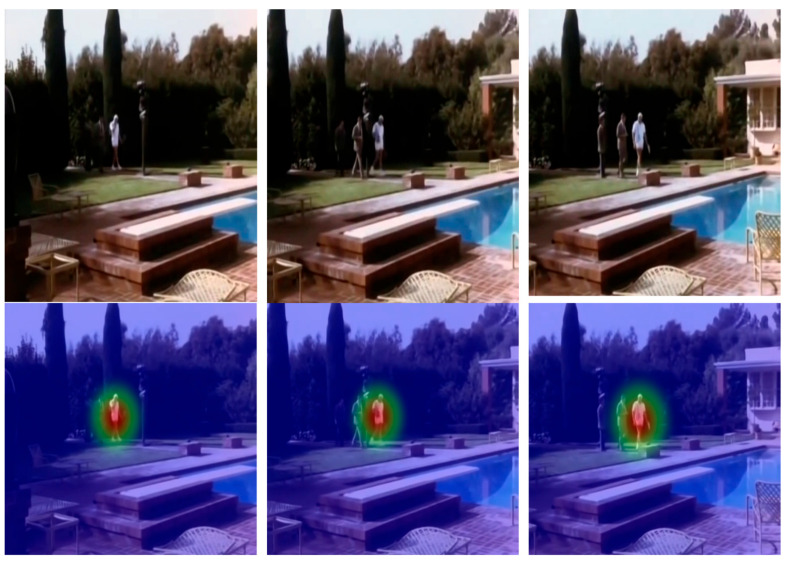
Visualization of confidence-based heatmaps generated by YOLO-Act for the action “walking”. The **top** row shows RGB frames sampled from different temporal positions (beginning, middle, end), while the **bottom** row shows the corresponding heatmaps overlaid on the same frames.

**Figure 8:**
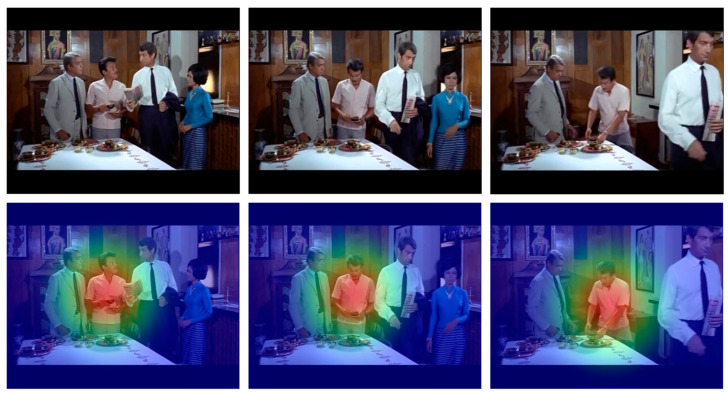
Visualization of confidence-based heatmaps generated by YOLO-Act for the action “putting down”. The **top** row shows RGB frames sampled from different temporal positions (beginning, middle, end), while the **bottom** row shows the corresponding heatmaps overlaid on the same frames.

While interpretability methods such as Grad-CAM are commonly used to visualize attention in convolutional neural networks, they are less suitable for YOLO-Act due to its underlying architecture. YOLO-Act is built upon the YOLO detection framework, which does not include explicit attention mechanisms or region proposal networks. Instead, it predicts bounding boxes and class probabilities directly from a grid of cells across the image, without generating spatial attention maps. Consequently, traditional attention visualization techniques like Grad-CAM cannot be directly applied. However, recognizing the importance of model interpretability and transparency, we propose the use of an alternative visualization strategy, namely confidence score heatmaps, which highlight the spatial regions where the model expresses the highest certainty. These heatmaps can serve as a lightweight and intuitive means to assess the model’s focus during inference. Thus, to provide an interpretable representation of YOLO-Act’s spatial reasoning, we generated confidence score heatmaps based on the grid cell activations within the YOLO detection head. Figure 7 shows a representative frame of a “walking” action. The semi-transparent overlay indicates the model’s internal confidence, with warmer colors (e.g., red, orange) denoting high-confidence regions and cooler colors (e.g., green, blue) indicating lower confidence. The model concentrates on the actor’s lower body, especially the moving legs. These regions are most indicative of the walking action. Alternatively, Figure 8 presents a frame from a “putting down” action. Here, the model focuses on the actor’s upper body, particularly the arms in motion. In both cases, although YOLO-Act lacks explicit attention modules, it implicitly identifies relevant spatial cues through its grid-based predictions. These confidence heatmaps provide a practical and effective alternative to traditional attention visualizations, enhancing interpretability and supporting model explainability in action recognition tasks.

The combination of precise spatial localization, temporal consistency through tracking, and explicit object modeling allows the YOLO-Act to address the key limitations of LART and other existing approaches. These innovations enable significant improvements across AVA subcategories, emphasizing the importance of integrating spatial and temporal reasoning in action recognition tasks.

**Figure 9:**
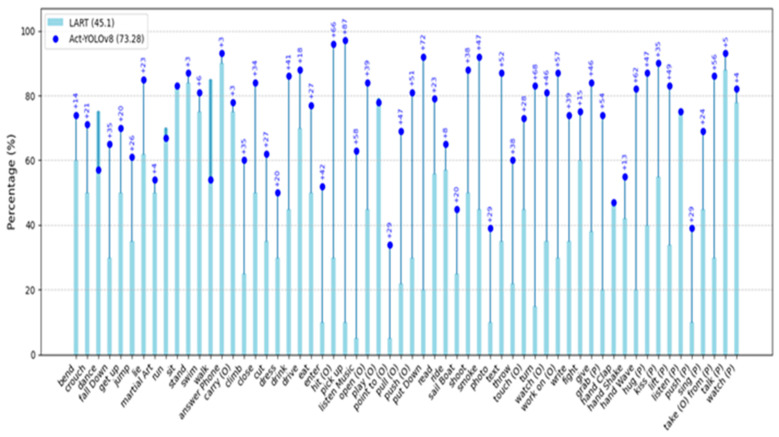
Comparison of YOLO-Act and LART [59] performances across action categories on AVA dataset.

Figure 9 highlights the performance of YOLO-Act compared with LART across 60 action classes in the AVA 2.2 dataset. The results demonstrate that YOLO-Act outperforms LART [59] in 54 out of 60 action categories, showcasing its robust capability in detecting and recognizing diverse human actions.

The model achieved substantial improvements in several challenging categories. For instance, it recorded a +72% improvement in the “pull up” class and an impressive +77% gain in the “push” class. Furthermore, the YOLO-Act demonstrates strong performance in actions involving object interactions, with notable gains such as +57% in “watch person”, +47% in “read”, and +42% in “talk to a person”. These results emphasize the ability of YOLO-Act to effectively capture complex interactions and utilize contextual information for accurate action recognition.

While YOLO-Act consistently outperforms LART, the magnitude of improvement varies across categories. For example, simpler or more ambiguous actions, such as “listening to music” and “eating”, exhibit smaller performance gains of +6% to +8%. These modest improvements may be attributed to limited training data or the inherent difficulty of distinguishing such actions because of minimal spatial-temporal cues.

Overall, the ability of YOLO-Act to integrate accurate spatial modeling with adequate temporal consistency enables it to excel across most action classes. Its advanced tracking mechanisms, robust bounding box predictions, and contextual aggregation significantly enhance performance, especially in categories requiring a nuanced understanding of human–object and human–human interactions. This consistent outperformance across a majority of action classes establishes YOLO-Act as a superior model for action detection.

## 5. Conclusions

The YOLO-Act is introduced in this paper to resolve the difficulties associated with detecting spatiotemporal human actions in videos. YOLO-Act extends YOLOv8 to action detection by introducing innovations such as keyframe-based temporal modeling and fusion strategies. The proposed model obtains substantial enhancements in computational efficiency and mAP compared with state-of-the-art methods, such as LART, as evidenced by experimental evaluations on the AVA dataset. The potential of the YOLO-Act for real-world applications, such as surveillance, human–computer interaction, and multimedia retrieval, is underscored by its capacity to precisely localize actions, capture temporal continuity, and manage complex interactions. Although YOLO-Act achieves strong performance in general action detection scenarios, its robustness under severe occlusion and rapid motion conditions remains unknown. In cases of heavy occlusion, the model may struggle to consistently track actors, leading to fragmented action recognition. Similarly, rapid movements can result in motion blur, which affects keyframe feature extraction accuracy. Potential strategies to alleviate these issues include the integration of motion-aware feature extractors, the adoption of occlusion-robust representation learning, and the incorporation of temporal attention mechanisms to better model dynamic visibility changes. Exploring these enhancements will form a key focus of future research to further improve YOLO-Act’s robustness across challenging real-world conditions. Future research may also investigate the integration of supplementary contextual indicators and the application of the YOLO-Act to broader multimodal scenarios that involve audio and textual data.

## Figures and Tables

**Table 2:** Summary of the advantages of deep learning approaches compared with manual methods.

Feature	Manual Methods	Deep Learning Methods
Feature Extraction	Hand-crafted, limited flexibility	Automatically learned from data
Adaptability	Poor generalization to new actions	High generalization capability
Temporal Modeling	Hand-tuned temporal descriptors	End-to-end learned temporal dynamics
Robustness	Sensitive to noise and occlusion	More robust through learned representations
Scalability	Difficult to extend to new classes	Easily scalable with additional data

**Table 3:** Comparison of attention-less and attention-based action recognition mechanisms.

Mechanism	Description	Advantages	Limitations
Attention-less Models	Rely solely on convolutional or temporal pooling operations without selective focus	Simpler, faster inference	May miss subtle or small-scale action cues
Attention-based Models	Use spatial, temporal, or spatio-temporal attention to prioritize important regions or frames	Higher accuracy, better interpretability	Increased computational complexity

**Table 4:** Comparison between YOLOv8 and the proposed YOLO-Act.

Feature	YOLOv8	YOLO-Act
Target	Static object detection	Dynamic action recognition
Temporal Modeling	None	Keyframe sequence modeling
Tracking	Not integrated	Integrated actor tracking
Late Fusion	Not applicable	Enhances multi-frame evidence
Application	Object detection tasks	Human action detection

**Table 5:** Training configurations.

Hyperparameters	Value
optimizer	AdamW
optimizer momentum	0.937
weight decay	0.0005
Learning rate schedule	Cosine Decay
warm-up epochs	3.0
drop out	0.1
learning rate	0.0001
batch size	32
epochs	100 early stopping at epoch 40
Patience/early stopping	10

**Table 6:** Performance comparison of YOLO-Act with state-of-the-art methods on AVA 2.2.

Model	mAP	FLOPs
SlowFast R101, 8×8 [25]	23.8	138
MViTv1-B, 64×3 [49]	27.3	296
SlowFast 16×8 +NL [25]	27.5	296
X3D-XL [48]	27.4	48
WOO [44]	28.3	251.7
MViTv1-B-24, 32×3 [49]	28.7	236
Object Transformer [54]	31.0	244
ACAR R101 [30]	33.3	435
MViT-L1312, 40×3 [50]	31.6	2828
MaskFeat [52]	39.8	2828
Video MAE [51]	42.6	241.61
Hiera [61]	43.3	1158
LART [59]	45.1	1260
YOLO-Act	73.28	114.41

**Table 7:** Performance comparison of YOLO-Act with LART [59] with respect to the three AVA subcategories: Object Manipulation (OM), Person Interactions (PI), and Person Movement (PM).

Model	OM	PI	PM	mAP
LART	37.3	45.7	63.8	45.1
YOLO-Act	76	74.9	67.1	73.28

**Table 8:** Module-level ablation study showing the contribution of the temporal aggregation module.

Model	Temporal Aggregation	mAP (%)	Performance Drop (%)
YOLO-Act	✓	73.28	-
Yolov8	✗	8.40	64.88

## Data Availability

Data are contained within the article.

## References

[1] Carreira J., Zisserman A. Quo Vadis, Action Recognition? A New Model and the Kinetics Dataset. Proceedings of the 2017 IEEE Conference on Computer Vision and Pattern Recognition (CVPR).

[2] Bah S.M., Ming F. (2020). An Improved Face Recognition Algorithm and Its Application in Attendance Management System. Array.

[3] Yang Q., Qin L., Chen Z., Ji S., Zhang K., Ma X. (2019). Empirical Study on the Impact of Short Video Content Marketing on Consumer’s Purchasing Intention Based on the Integrated Model of TRA and ELM. Proceedings of the 1st International Symposium on Economic Development and Management Innovation (EDMI 2019).

[4] Esteva A., Chou K., Yeung S., Naik N., Madani A., Mottaghi A., Liu Y., Topol E., Dean J., Socher R. (2021). Deep Learning-Enabled Medical Computer Vision. NPJ Digit. Med..

[5] Chao G.-C., Tsai Y.-P., Jeng S.-K. (2010). Augmented 3-D Keyframe Extraction for Surveillance Videos. IEEE Trans. Circuits Syst. Video Technol..

[6] Pérez-Hernández F., Tabik S., Lamas A., Olmos R., Fujita H., Herrera F. (2020). Object Detection Binary Classifiers Methodology Based on Deep Learning to Identify Small Objects Handled Similarly: Application in Video Surveillance. Knowl. Based Syst..

[7] Lu X., Yao H., Zhao S., Sun X., Zhang S. (2019). Action Recognition with Multi-Scale Trajectory-Pooled 3D Convolutional Descriptors. Multimed. Tools Appl..

[8] Bhoi A. (2019). Spatio-Temporal Action Recognition: A Survey. arXiv.

[9] Pareek P., Thakkar A. (2021). A Survey on Video-Based Human Action Recognition: Recent Updates, Datasets, Challenges, and Applications. Artif. Intell. Rev..

[10] Jiao L., Zhang R., Liu F., Yang S., Hou B., Li L., Tang X. (2022). New Generation Deep Learning for Video Object Detection: A Survey. IEEE Trans. Neural Netw. Learn. Syst..

[11] Pouyanfar S., Sadiq S., Yan Y., Tian H., Tao Y., Reyes M.P., Shyu M.L., Chen S.C., Iyengar S.S. (2018). A Survey on Deep Learning: Algorithms, Techniques, and Applications. ACM Comput. Surv..

[12] Karpathy A., Toderici G., Shetty S., Leung T., Sukthankar R., Fei-Fei L. Large-Scale Video Classification with Convolutional Neural Networks. Proceedings of the 2014 IEEE Conference on Computer Vision and Pattern Recognition (CVPR).

[13] Pramanik A., Pal S.K., Maiti J., Mitra P. (2021). Granulated RCNN and Multi-Class Deep SORT for Multi-Object Detection and Tracking. IEEE Trans. Emerg. Top. Comput. Intell..

[14] Simonyan K., Zisserman A. (2019). Two-Stream Convolutional Networks for Action Recognition in Videos. arXiv.

[15] Tran D., Bourdev L., Fergus R., Torresani L., Paluri M. Learning Spatiotemporal Features with 3d Convolutional Networks. Proceedings of the 2015 IEEE International Conference on Computer Vision (ICCV).

[16] Ji S., Xu W., Yang M., Yu K. (2012). 3D Convolutional Neural Networks for Human Action Recognition. IEEE Trans. Pattern Anal. Mach. Intell..

[17] Murthy C.B., Hashmi M.F., Bokde N.D., Geem Z.W. (2020). Investigations of Object Detection in Images/Videos Using Various Deep Learning Techniques and Embedded Platforms-A Comprehensive Review. Appl. Sci. Switz..

[18] Wang X., Girshick R., Gupta A., He K. Non-Local Neural Networks. Proceedings of the 2017 IEEE Conference on Computer Vision and Pattern Recognition (CVPR).

[19] Wu C.-Y., Feichtenhofer C., Fan H., He K., Krähenbühl P., Girshick R. Long-Term Feature Banks for Detailed Video Understanding. Proceedings of the 2019 IEEE/CVF Conference on Computer Vision and Pattern Recognition (CVPR).

[20] Hu J., Shen L., Sun G. Squeeze-and-Excitation Networks. Proceedings of the 2017 IEEE Conference on Computer Vision and Pattern Recognition (CVPR).

[21] Dollar P., Rabaud V., Cottrell G., Belongie S. Behavior Recognition via Sparse Spatio-Temporal Features. Proceedings of the 2005 IEEE International Workshop on Visual Surveillance and Performance Evaluation of Tracking and Surveillance.

[22] Klaser A., Marszałek M., Schmid C. (2008). A Spatio-Temporal Descriptor Based on 3D-Gradients. Proceedings of the BMVC 2008-19th British Machine Vision Conference.

[23] Wang H., Schmid C. Action Recognition with Improved Trajectories. Proceedings of the 2013 IEEE International Conference on Computer Vision.

[24] Sevilla-Lara L., Liao Y., Güney F., Jampani V., Geiger A., Black M.J., Brox T., Bruhn A., Fritz M. (2019). On the Integration of Optical Flow and Action Recognition.

[25] Feichtenhofer C., Fan H., Malik J., He K. SlowFast Networks for Video Recognition. Proceedings of the 2019 IEEE/CVF International Conference on Computer Vision (ICCV).

[26] Wang X., Gupta A. Videos as Space-Time Region Graphs. Proceedings of the European Conference on Computer Vision (ECCV).

[27] Gao C., Xu J., Zou Y., Huang J.-B., Vedaldi A., Bischof H., Brox T., Frahm J.-M. (2020). DRG: Dual Relation Graph for Human-Object Interaction Detection.

[28] Sun C., Shrivastava A., Vondrick C., Sukthankar R., Murphy K., Schmid C. Relational Action Forecasting. Proceedings of the 2019 IEEE/CVF Conference on Computer Vision and Pattern Recognition.

[29] Tang J., Xia J., Mu X., Pang B., Lu C. (2020). Asynchronous Interaction Aggregation for Action Detection. arXiv.

[30] Pan J., Chen S., Shou M.Z., Liu Y., Shao J., Li H. Actor-Context-Actor Relation Network for Spatio-Temporal Action Localization. Proceedings of the 2021 IEEE/CVF Conference on Computer Vision and Pattern Recognition (CVPR).

[31] Rani M., Kumar M. (2025). MobileNet for Human Activity Recognition in Smart Surveillance Using Transfer Learning. Neural Comput. Appl..

[32] Piergiovanni A.J., Angelova A., Ryoo M.S. (2022). Tiny Video Networks. Appl. AI Lett..

[33] Deep Learning Approach to Key Frame Detection in Human Action Videos. https://www.intechopen.com/chapters/71081.

[34] Arslan S., Tanberk S. (2023). Key Frame Extraction with Attention Based Deep Neural Networks. arXiv.

[35] Zhang Y., Wang C., Wang X., Zeng W., Liu W. (2021). Fairmot: On the Fairness of Detection and Re-Identification in Multiple Object Tracking. Int. J. Comput. Vis..

[36] Yue J., Ng H., Hausknecht M., Vijayanarasimhan S., Vinyals O., Monga R., Toderici G. Beyond Short Snippets: Deep Networks for Video Classification. Proceedings of the 2015 IEEE Conference on Computer Vision and Pattern Recognition (CVPR).

[37] Rumelhart D.E., Hinton G.E., Williams R.J. (1986). Learning Representations by Back-Propagating Errors. Nature.

[38] Hochreiter S., Schmidhuber J. (1997). Long Short-Term Memory. Neural Comput..

[39] Cui Y., Jia M., Lin T.-Y., Song Y., Belongie S. Class-Balanced Loss Based on Effective Number of Samples. Proceedings of the 2019 IEEE/CVF Conference on Computer Vision and Pattern Recognition.

[40] Pulakurthi P.R., De Melo C.M., Rao R., Rabbani M. (2024). Enhancing Human Action Recognition with GAN-Based Data Augmentation. Proceedings of the Synthetic Data for Artificial Intelligence and Machine Learning: Tools, Techniques, and Applications II.

[41] Alshdaifat N.F.F., Talib A.Z., Osman M.A. (2020). Improved Deep Learning Framework for Fish Segmentation in Underwater Videos. Ecol. Inform..

[42] He K., Zhang X., Ren S., Sun J. (2015). Deep Residual Learning for Image Recognition. arXiv.

[43] Krizhevsky A., Sutskever I., Hinton G.E. (2012). Imagenet Classification with Deep Convolutional Neural Networks. Adv. Neural Inf. Process. Syst..

[44] Chen S., Sun P., Xie E., Ge C., Wu J., Ma L., Shen J., Luo P. Watch Only Once: An End-to-End Video Action Detection Framework. Proceedings of the 2021 IEEE/CVF International Conference on Computer Vision (ICCV).

[45] Wu Y., Zhao Z., Chen P., Guo F., Qin Y., Long S., Ai L. (2025). Hybrid Learning Architecture for High-Speed Railroad Scene Parsing and Potential Safety Hazard Evaluation of UAV Images. Measurement.

[46] Chen Z., Yang J., Feng Z., Zhu H. (2024). RailFOD23: A Dataset for Foreign Object Detection on Railroad Transmission Lines. Sci. Data.

[47] Yan L., Wang Q., Zhao J., Guan Q., Tang Z., Zhang J., Liu D., Leonardis A., Ricci E., Roth S., Russakovsky O., Sattler T., Varol G. (2025). Radiance Field Learners As UAV First-Person Viewers. Computer Vision–ECCV 2024.

[48] Feichtenhofer C. X3D: Expanding Architectures for Efficient Video Recognition. Proceedings of the 2020 IEEE/CVF Conference on Computer Vision and Pattern Recognition (CVPR).

[49] Fan H., Xiong B., Mangalam K., Li Y., Yan Z., Malik J., Feichtenhofer C. Multiscale Vision Transformers. Proceedings of the 2021 IEEE/CVF International Conference on Computer Vision.

[50] Li Y., Wu C.-Y., Fan H., Mangalam K., Xiong B., Malik J., Feichtenhofer C. MViTv2: Improved Multiscale Vision Transformers for Classification and Detection. Proceedings of the 2022 IEEE/CVF Conference on Computer Vision and Pattern Recognition.

[51] Tong Z., Song Y., Wang J., Wang L. (2022). VideoMAE: Masked Autoencoders Are Data-Efficient Learners for Self-Supervised Video Pre-Training. Adv. Neural Inf. Process Syst..

[52] Wei C., Fan H., Xie S., Wu C.-Y., Yuille A., Feichtenhofer C. Masked Feature Prediction for Self-Supervised Visual Pre-Training. Proceedings of the 2022 IEEE/CVF Conference on Computer Vision and Pattern Recognition (CVPR).

[53] He K., Chen X., Xie S., Li Y., Dollár P., Girshick R. Masked Autoencoders Are Scalable Vision Learners. Proceedings of the 2022 IEEE/CVF Conference on Computer Vision and Pattern Recognition.

[54] Wu C.-Y., Krahenbuhl P. Towards Long-Form Video Understanding. Proceedings of the 2021 IEEE/CVF Conference on Computer Vision and Pattern Recognition.

[55] Vo-Ho V.-K., Le N., Yamazaki K., Sugimoto A., Tran M.-T. Agent-Environment Network for Temporal Action Proposal Generation. Proceedings of the ICASSP 2021 - 2021 IEEE International Conference on Acoustics, Speech and Signal Processing (ICASSP).

[56] Köpüklü O., Wei X., Rigoll G. (2019). You Only Watch Once: A Unified CNN Architecture for Real-Time Spatiotemporal Action Localization. arXiv.

[57] Redmon J., Farhadi A. YOLO9000: Better, Faster, Stronger. Proceedings of the 2017 IEEE Conference on Computer Vision and Pattern Recognition.

[58] Hara K., Kataoka H., Satoh Y. Can Spatiotemporal 3D CNNs Retrace the History of 2D CNNs and ImageNet?. Proceedings of the 2018 IEEE Conference on Computer Vision and Pattern Recognition.

[59] Rajasegaran J., Pavlakos G., Kanazawa A., Feichtenhofer C., Malik J. On the Benefits of 3D Pose and Tracking for Human Action Recognition. Proceedings of the 2023 IEEE/CVF Conference on Computer Vision and Pattern Recognition.

[60] Rajasegaran J., Pavlakos G., Kanazawa A., Malik J. Tracking People by Predicting 3D Appearance, Location and Pose. Proceedings of the 2022 IEEE/CVF Conference on Computer Vision and Pattern Recognition.

[61] Ryali C., Hu Y.-T., Bolya D., Wei C., Fan H., Huang P.-Y., Aggarwal V., Chowdhury A., Poursaeed O., Hoffman J. (2023). Hiera: A Hierarchical Vision Transformer without the Bells-and-Whistles. Proceedings of the International Conference on Machine Learning.

[62] Ren S., He K., Girshick R., Sun J. (2015). Faster R-Cnn: Towards Real-Time Object Detection with Region Proposal Networks. Adv. Neural Inf. Process Syst..

[63] Gu C., Sun C., Ross D.A., Vondrick C., Pantofaru C., Li Y., Vijayanarasimhan S., Toderici G., Ricco S., Sukthankar R. AVA: A Video Dataset of Spatio-Temporally Localized Atomic Visual Actions. Proceedings of the 2018 IEEE/CVF Conference on Computer Vision and Pattern Recognition.

[64] Soomro K., Zamir A.R., Shah M. (2012). UCF101: A Dataset of 101 Human Actions Classes from Videos in the Wild. arXiv.

[65] Jhuang H., Gall J., Zuffi S., Schmid C., Black M.J. Towards Understanding Action Recognition. Proceedings of the 2013 IEEE International Conference on Computer Vision.

[66] Heilbron F.C., Escorcia V., Ghanem B., Niebles J.C. (2015). Activitynet: A Large-Scale Video Benchmark for Human Activity Understanding. Proceedings of the 2015 IEEE Conference on Computer Vision and Pattern Recognition (CVPR).

[67] Idrees H., Zamir A.R., Jiang Y.-G., Gorban A., Laptev I., Sukthankar R., Shah M. (2017). The Thumos Challenge on Action Recognition for Videos “in the Wild”. Comput. Vis. Image Underst..

[68] Jocher G., Chaurasia A., Qiu J. (2023). YOLO by Ultralytics (Version 8.0.0). https://github.com/ultralytics/ultralytics.

[69] Lin T.-Y., Maire M., Belongie S., Bourdev L., Girshick R., Hays J., Perona P., Ramanan D., Zitnick C.L., Dollár P. Microsoft COCO: Common Objects in Context. Proceedings of the Computer Vision–ECCV 2014: 13th European Conference.

[70] Girdhar R., Carreira J., Doersch C., Zisserman A. (2018). A Better Baseline for AVA. arXiv.

[71] Zhao J., Zhang Y., Li X., Chen H., Bing S., Xu M., Liu C., Kundu K., Xiong Y., Modolo D. TubeR: Tubelet Transformer for Video Action Detection. Proceedings of the 2022 IEEE/CVF Conference on Computer Vision and Pattern Recognition (CVPR).

